# What Young People Want From a Sexual Health Website: Design and Development of Sexunzipped

**DOI:** 10.2196/jmir.2116

**Published:** 2012-10-12

**Authors:** Ona McCarthy, Kenneth Carswell, Elizabeth Murray, Caroline Free, Fiona Stevenson, Julia V Bailey

**Affiliations:** ^1^e-Health UnitResearch Department of Primary Care & Population HealthUniversity College LondonLondonUnited Kingdom; ^2^Barts & The London School of Medicine and DentistryQueen Mary, University of LondonLondonUnited Kingdom; ^3^Department of Nutrition and Public Health Intervention ResearchLondon School of Hygiene & Tropical MedicineLondonUnited Kingdom

**Keywords:** Sex education, adolescents, young adults, qualitative research

## Abstract

**Background:**

Sexual health education in the United Kingdom is of variable quality, typically focusing on the biological aspects of sex rather than on communication, relationships, and sexual pleasure. The Internet offers a unique opportunity to provide sexual health education to young people, since they can be difficult to engage but frequently use the Internet as a health information resource.

**Objectives:**

To explore through qualitative research young people’s views on what elements of a sexual health website would be appealing and engaging, and their views on the content, design, and interactive features of the Sexunzipped intervention website.

**Methods:**

We recruited 67 young people aged 16–22 years in London, UK. We held 21 focus groups and 6 one-to-one interviews to establish sexual health priorities, views on website look and feel, and what features of a sexual heath website would attract and engage them. Two researchers facilitated the focus groups, using a semistructured topic guide to lead the discussions and asking open questions to elicit a range of views. The discussions and interviews were audio recorded and detailed notes were made on key topics from the audio recording. Young people’s views influenced design templates for the content and interactive features of Sexunzipped.

**Results:**

Young people particularly wanted straightforward information on sexual pleasure, sexually transmitted infections and pregnancy, how to communicate with partners, how to develop skills in giving pleasure, and emotions involved in sex and relationships. Focus group participants wanted social interaction with other young people online and wanted to see themselves reflected in some way such as through images or videos.

**Conclusions:**

While it is challenging to meet all of young people’s technological and design requirements, consultation with the target audience is valuable and necessary in developing an online sexual health intervention. Young people are willing to talk about sensitive issues, enjoy the discussions, and can offer key insights that influence intervention development.

## Introduction

Sexual health information provided in UK schools is taught within the statutory requirements of the National Science Curriculum [1]. It is variable in quality and tends to focus on the biological facts about reproduction, condom use, and sexually transmitted infections [1]. Consequently, information on skills building, sexual communication, relationship decision making, and sexual pleasure are generally missing from young people’s sexual learning experience. Including these elements in young people’s sexual education would align with one definition of sexual health put forth by the World Health Organization:

*...a state of physical, emotional, mental and social well-being in relation to sexuality; it is not merely the absence of disease, dysfunction or infirmity. Sexual health requires a positive and respectful approach to sexuality and sexual relationships, as well as the possibility of having pleasurable and safe sexual experiences, free of coercion, discrimination and violence. For sexual health to be attained and maintained, the sexual rights of all persons must be respected, protected and fulfilled. [2]*

Young people can be seen as a hard-to-reach group for health promotion. Internet interventions may be one way of reaching young people, as they frequently use the Internet to search for health information [3-5] and appreciate the privacy, convenience, and ease of accessing information on the Web [4,5]. These potential advantages are particularly valued for stigmatized or sensitive subjects such as sexual health. A further potential advantage of using the Internet for sexual health promotion is the relative ease of covering a broad range of information including sexual pleasure. Some argue that, since one of the main motivations for engaging in sex is pleasure, pleasure must be addressed in the discourse of sexual health promotion [6]. A systematic review of interactive computer-based interventions found that they can help increase sexual knowledge and have a positive effect on safer-sex self-efficacy, intention, and behavior [7].

To determine the feasibility of promoting sexual health on the Internet to young people, we first set out to determine young people’s views about whether an Internet intervention could help meet their sexual health needs and, if so, how best this could be done. This paper reports on our findings and on how they informed the development of an Internet intervention for sexual health known as Sexunzipped [8]. Additional details on integrating psychological theory into the design of this online intervention have been described elsewhere [9].

## Methods

### Design and Setting

We conducted 21 focus groups in London, UK, at young people’s community sexual health clinics and held 6 one-to-one interviews in the Research Department of Primary Care and Population Health, University College London. Ethical approval was obtained from the University College London Research Ethics Committee (ref number 1023/001).

### Participants

Young people aged 16–22 years were eligible to participate in the study.

### Recruitment

We used multiple methods of recruitment, including approaching young people in sexual health clinic waiting rooms, distributing flyers around clinics and colleges, approaching key contacts involved in youth work, and placing advertisements on youth websites such as the UK Youth Parliament and college webpages. Snowballing was also used, where initial participants were asked to recruit potentially interested friends.

Participants were initially asked to attend only 1 focus group but were invited to subsequent groups if they were particularly engaged. Repeated attendance at focus groups over time allowed these participants to build on previous discussions. They were able to comment on refinements made to earlier website content and interactive formats.

We asked 3 focus group participants who were particularly engaged to attend a one-to-one interview. We also recruited 3 interviewees from online advertisements on youth websites and through key youth work contacts.

All participants provided written consent to their participation before attending a focus group or interview and were offered a £10 (US $16) cash incentive if they stayed for the duration.

### Data Collection

The focus group discussions were used to explore (1) young people’s sexual health priorities, (2) their views on website look and feel, and (3) what features of an interactive sexual heath website would attract and engage them.

All focus groups were moderated by 2 facilitators who introduced the project, explained the purpose of the focus group, set ground rules of mutual respect and confidentiality, and ensured that all participants had provided written informed consent. Participants provided anonymous demographic information by completing a paper form. The discussions and interviews were audio recorded with participants’ permission.

Semistructured topic guides were used to guide the focus group discussions. These guides evolved as data were collected. Open-ended questioning was used to elicit a wide range of views, and participants were asked to elaborate on relevant topics beyond the topic guide. Less-vocal participants were encouraged to share their views. We developed the Sexunzipped website in parallel with the emerging data from the focus groups, with early website design templates prompting discussion in subsequent focus groups. This enabled participants to comment on the presentation and content of the intervention, leading to iterative changes as a form of participatory design. Focus group discussions ranged from 60 to 90 minutes, depending on the quality of the information gathered and participants’ attention spans.

One-to-one interviews lasted 60 minutes and used open-ended questioning. The interviews were used to gather views on early versions of website content and to generate anonymous quotations on sexual health issues to use throughout the website. To test the website content, we asked participants to read and comment on sample content and to give feedback on paper versions of interactive activities. To generate the quotations, the interviewer asked participants for their views on potentially controversial sex-related topics, such as “What do you think about girls carrying condoms?”. Anonymous quotations for the website were also generated by adapting comments taken from youth website discussion boards.

### Data Analysis

One researcher made detailed notes on key themes from the focus group audio recordings. The analytic notes were reviewed and discussed by the core research team consisting of a clinical psychologist, a health services researcher, and a sexual health clinician. Key findings were documented, and emergent themes were tested in subsequent focus groups until the core team was satisfied that the information gained was sufficient. Information from the one-to-one content testing and quotation generation was used to refine subsequent website content.

## Results

### Participant Characteristics

A total of 67 people aged 16–22 years living in the United Kingdom participated in 21 focus groups and 6 one-to-one interviews over 16 months (Table 1). We used 3 one-to-one interviews to test website content and 3 to generate anonymous quotations for use throughout the website.

Of the 67 participants, 48 (72%) were aged 16–17 years, and 50 (75%) were female. Participants were ethnically diverse, with 26 (39%) describing themselves as white British or white other, 26 (39%) as black British, 2 (3%) Asian British, 7 (10%) as mixed, and 6 (9%) as other. The median age at which they first had sex was 15 years for both young women and men, with 33 (66%) young women ever having had sex and 15 (88%) young men ever having had sex. Of the 67 participants, 57 (85%) were in education or training. Most participants lived in inner London, with 2 living outside but near London. Of the 67 participants, 20 (30%) young people participated more than once.

**Table 1:** Self-reported participant characteristics (n = 67).

Demographic characteristic	n	%	
**Age (years)**			
	16–17	48	72%
	18–22	19	28%
**Gender**			
	Female	50	75%
	Male	17	25%
**Ever had sex**			
	Yes	48	72%
	No	19	28%
**Ethnic group**			
	White British/white other	26	39%
	Black British	26	39%
	Asian British	2	3%
	Mixed	7	10%
	Other	6	9%
**Education/work**			
	In education or training	57	85%
	Working	3	4%
	Other	7	10%

### Young People’s Views on the Creation of a Sexual Health Website

Focus group participants appreciated being consulted and were eager to share their views. They supported the creation of a sexual health website with input from young people because they felt that current sites did not fully address their needs. They provided a wealth of information about their sexual health priorities and what features of such a website would be attractive and engaging to them.

#### Content

Participants wanted straightforward information on sexual pleasure, sexually transmitted infections and pregnancy, how to communicate with partners, how to develop skills in giving pleasure, and emotions involved in sex and relationships.

Young people wanted information on the pleasurable aspects of sex, presented in a nonjudgmental way (eg, it’s acceptable to have casual sex as long as both people consent and are happy about it) while recognizing the importance of more serious, risk-focused sexual health topics. They were enthusiastic about including information on masturbation, and sexual positions and practices, and wanted general tips on how to be a better lover. They also wanted to hear others’ experiences about giving and getting pleasure.

Participants wanted help with developing skills in communicating about sex and dealing with emotions within sexual relationships. They also wanted practical tips on how to communicate about sensitive issues such as negotiating condom use and talking about what kind of sex they do or do not like.

Participants thought a sexual health website should include information relevant for young people at different stages of sexual experience and exploration. They suggested it should cover information and activities for those who had not yet had sex, such as information on sex for the first time and advice on how to avoid unwanted events, as well as content for those who are looking to explore new sexual practices. Participants felt inclusion of more advanced sexual practices such as anal sex and fisting would not be intimidating or create feelings of inadequacy as long as content was balanced with other sexual health information. They speculated that advanced sexual content would attract young people to the website because such content is usually absent from youth-targeted resources, and if users were not interested in certain areas of the website, they simply would not read it.

Young people wanted website content presented in a straightforward style that sounds honest, accurate, and not “preachy.” They recommended this style should not sound too serious and should not come across as “school work.” They preferred content written in the voice of someone credible and knowledgeable but also someone they can relate to and respect such as an older sibling.

They preferred the use of uncomplicated words and phrases, for example, sex instead of sexual intercourse. For those situations where an uncomplicated word is unavailable, they suggested providing a definition, such as “transmit = to pass on to someone.” They were also adamant that “youth-speak” or slang (eg, coz for because) should not be used, as the website would come across as trying too hard, although young people felt that it could sometimes be appropriate to use simpler versions of words such as lube for lubrication and come for ejaculate.

Young people did not want what they interpreted as clichés such as “I feel like I should be having sex because everyone else is” included on the site. They recognized an existence of double standards for men and women regarding sex (eg, it’s acceptable for men to have numerous partners but not for women) and valued a space to address these issues.

#### Website Look and Feel

There was no clear consensus on website colors and logo design, with some preferring bold colors and others neutral tones. Participants wanted images of people, scenarios, and “real-life stuff” they could relate to such as young people partying in clubs, kissing, and hanging out on the street. They felt that images of young people should reflect the diversity of the UK population.

Participants wanted a website to include specific sexual health images such as photographs of sexually transmitted infections and contraception options. Young people expected the website logo to reflect sex and not require a lot of thought to understand its significance. They suggested the website name should be clear, be memorable, and suggest the content of the website. Young people wanted the site to feel active, with new activities and information added each week. They disliked pages containing a lot of text, which they said that they would not read.

#### Engaging Website Features

Focus group participants wanted social interaction with other young people such as through discussion boards. A post-and-comment format used on social networking websites such as Facebook was a popular suggestion, since it is familiar and enables users to interact. Young people said that all communication must be anonymous so as not to disclose their identity if “embarrassing things” were posted.

Young people wanted videos to feature on a sexual health website but specified that these videos must represent real people talking about real experiences. If this is impossible, high-quality actors must be used; if the people and situations in videos are not believable and realistic, the young people would not relate to them and would not find them valuable. One suggestion was to include short clips of young people sharing real stories on a range of issues with a means of commenting on the videos such as on the website YouTube.

Another feature that appealed to young people was a dramatic story format in which the user can choose different courses of action at pivotal points in the narrative. Participants liked the idea that dramas could have different endings depending on what decisions they made along the way and said they would be disappointed if they received the same feedback after different story endings.

### Design and Content of the Sexunzipped Website

Paying particular attention to young people’s desire for interactivity, we integrated young people’s views with psychological theories of behavior change to create the Sexunzipped theory-based intervention website [9]. Reflecting young people’s suggestions and priorities, the website content is divided into three major sections: Relationships, Safer Sex, and Sexual Pleasure. Each section contains a combination of interactive quizzes and decision-making activities, as well as text-based information. Links to other related topics on the site are provided at the end of all activities and text-based pages.

#### Information Pages

**Figure 1:**
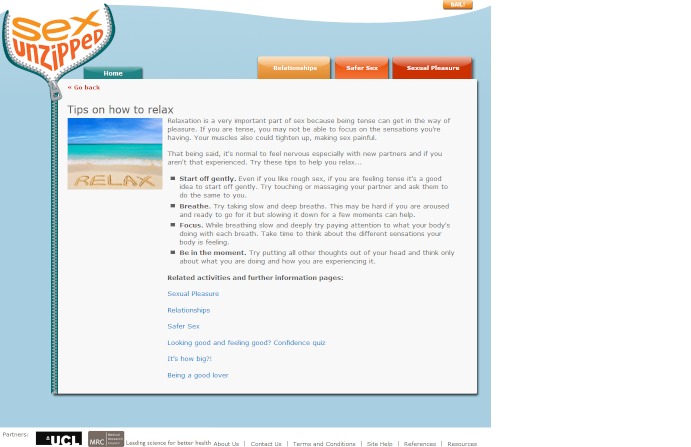
Example information page on the Sexunzipped website.

The text-based information pages throughout each of the three sections cover the major issues raised by the young people under the headings Relationships, Safer Sex, and Sexual Pleasure (Figure 1).

#### Interactive Quizzes

**Figure 2:**
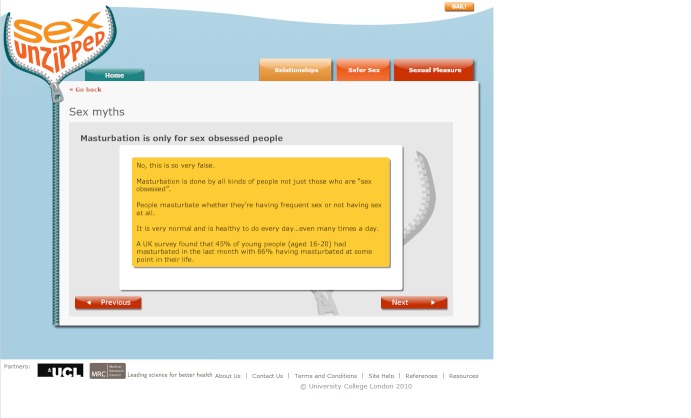
Interactive quiz: example feedback.

Drawing from behavior change theory [9], the quizzes on Sexunzipped were designed to prompt active learning and reflection by providing feedback according to responses given by users (Figure 2). Response options were designed to encourage users to reflect on their views, emotions, and experiences, as well as providing information on social norms, and encouraging beliefs and attitudes associated with safer-sex behavior. Incorporating what we learned from the focus groups, we designed and wrote the quizzes to be entertaining as well as educational, keeping in mind that young people said they valued an honest tone that does not “try too hard.”

#### Interactive Decision-Making Activities

**Figure 3:**
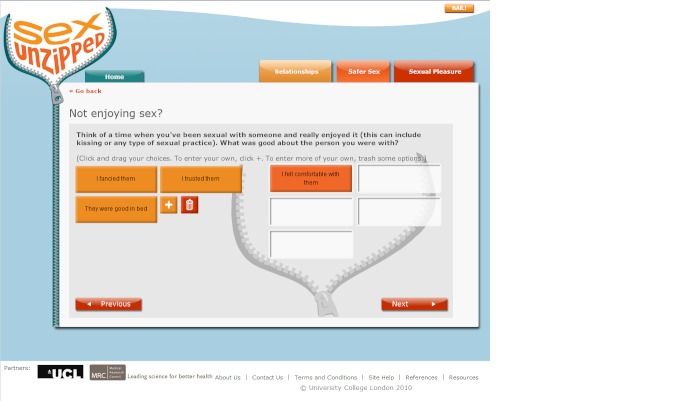
Decision-making activity: example question with drag-and-drop feature.

The decision-making activities were designed to provoke self-reflection about sexual behavior, focusing on problematic situations or dilemmas concerning relationships, or risky or regretted sexual situations (Figure 3). Participants had strong feelings about the length of these activities, which they said must be short and not require a lot of writing. To address this, we created a format whereby users could drag and drop prespecified response options but could also add their own.

**Figure 4:**
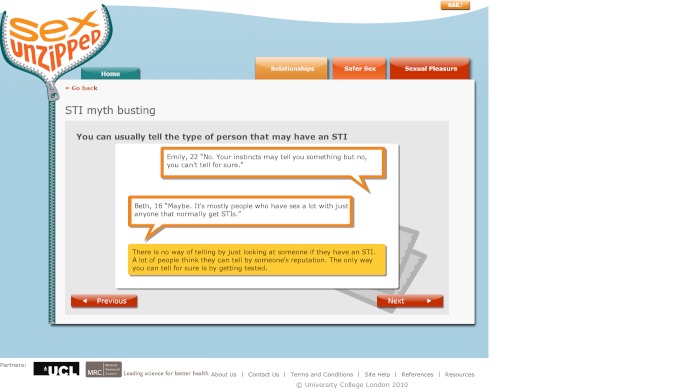
Example quotations regarding sexually transmitted infection (STI).

The website also includes quotations with aliases reflecting a range of views intending to give the feel of a peer-to-peer exchange of views (Figure 4).

## Discussion

### Principal Results

Participants were actively engaged in the focus group discussions and enjoyed taking part. They offered key insights into the content, look, and feel and interactive features of the Sexunzipped sexual health website. The resulting intervention has been tested in a pilot online trial (trial registration number ISRCTN55651027).

The strongest message to emerge from this research was the importance of providing young people with honest sexual health information that features content on sexual pleasure and relationships. Participants desired a website that reflects a mature, trustworthy, and true-to-life feel, conveyed through the voice of a knowledgeable youth worker or older sibling. The need for the website to appear trustworthy [10,11] along with the potential to share and compare real-life experiences [12] is consistent with previous findings. Our focus group findings align with other research, which found that young people are interested in receiving sexual health information through digital technologies such as the Internet and mobile phones [11,13], and are interested in knowing more about sexual pleasure, feelings, and emotions [14].

The integration of pleasure and relationship information with traditional risk-focused sexual health promotion was seen as novel and attractive, with participants preferring this approach to their experiences of sexual health education delivered by schools or by health authorities. Giving equal weight to traditional risk-based messages and sexual pleasure (including masturbation, for example) would give the message that both are important for healthy sexual lives [15,16].

A wide variety of sexual health websites targeted at young people are available on the Internet, for example, those developed in consultation with young people [17], those including information on sexual pleasure [18,19] and those offering multimedia features such as videos of people talking about their experiences [20,21]. The Sexunzipped website combines these elements in that it was developed with young people, features sexual pleasure, and includes theory-based interactive activities.

### Addressing What Young People Want in a Website

Perhaps the greatest challenge was to integrate young people’s preferences with psychological theory [9] within technical and budgetary constraints to create an acceptable and engaging sexual health website.

Participants expressed their desire for direct social interaction with other young people online (eg, via discussion boards and instant messaging). These features are expensive, require moderation, and raise ethical issues, for example, regarding online bullying, or disclosure of illegal or dangerous activities. There was also a risk that if no one used the discussion board, the site would appear unpopular and therefore unattractive. Young people wanted to see themselves reflected on the site (eg, in videos and drama), but this is expensive to produce and is difficult to pitch correctly. Young people are accustomed to the actively changing landscape on the Internet and wanted new activities and information added on a regular basis, which is resource intensive.

Consultation with users is essential in the development of websites targeted at young people, as this group can be particularly influenced by look and feel. This research and previous research suggest that listening to and meeting young people’s desires in terms of website design and content is essential in engaging them [10,12]. However, websites may quickly look and feel out-of-date due to the rapidly changing online environment, which necessitates regular user consultation and updating. This requires careful financial planning by those involved in site maintenance in order to support the continued user consultation and updating.

### Limitations

Focus group participants were London-based young people, which could mean that any differences in sexual health priorities due to geographical location may be underrepresented in the content of Sexunzipped. However, we thought that young people in other areas of the United Kingdom would be able to recognize and relate to inner-city youth culture to some degree. The Sexunzipped website design was intended to appeal to a broad range of young people, avoiding any specific styles that could alienate particular groups or become quickly out-of-date.

The sample contained 75% (50/67) women because greater numbers of young women attend community sexual health clinics. This may have resulted in views more representative of female needs and priorities. We made attempts to include more young men such as contacting male youth organizations, but this group proved to be less accessible than young women.

Although most focus groups included participants who did not know each other, the snowballing method of recruitment meant that the sample could have resulted in participants having similar views.

### Conclusions

Consultation with the target audience is valuable and necessary in developing sexual health interventions, particularly in developing interventions for young people. It can be difficult to pitch content correctly, but it is possible with repeated consultation with users. It is challenging to incorporate all of young people’s technological desires, but digital technology makes it possible to create an engaging online intervention that includes a variety of interactive formats covering a broad range of topics. Young people are willing to talk about sensitive issues, enjoy the discussions, and can offer key insights that influence intervention development.
